# The Influence of Two-Region Morphology and Grain Shape on the Transport Critical Current Density in the Range from 15 K to 30 K in SiC-Doped MgB_2_ Wires Fabricated by the Powder-in-Tube Method

**DOI:** 10.3390/ma18173960

**Published:** 2025-08-24

**Authors:** Daniel Gajda, Michał Babij, Andrzej Zaleski, Dogan Avci, Hakan Yetis, Ibrahim Belenli, Fırat Karaboga, Damian Szymanski, Tomasz Czujko

**Affiliations:** 1 Institute of Low Temperature and Structure Research, Polish Academy of Sciences (PAS), 50-422 Wroclaw, Poland; m.babij@intibs.pl (M.B.); a.zaleski@intibs.pl (A.Z.); d.szymanski@intibis.pl (D.S.); 2 Quatum Metrology Laboratory, National Metrology Institute TÜB ITAK, 41470 Kocaeli, Türkiye; davci.0209@gmail.com; 3 Department of Physics, Bolu Abant Izzet Baysal University, 14280 Bolu, Türkiye; hknyetis@gmail.com (H.Y.); belenli_i@ibu.edu.tr (I.B.); 4 Mehmet Tanrikulu Vocational School of Health Services, Bolu Abant Izzet Baysal University, 14030 Bolu, Türkiye; karabogafirat@ibu.edu.tr; 5 Institute of Materials Science and Engineering, Military University of Technology, 00-908 Warsaw, Poland

**Keywords:** MgB_2_, superconductive wires, critical current density

## Abstract

The paper presents the results of the influence of SiC dopant, annealing temperature, and annealing time on the morphology of MgB_2_ material in superconducting wires. The results of measurements of critical temperature (*T*_c_), irreversible magnetic field (*B*_irr_), resistance in the normal state (*R*_n_), and transport critical current density (*J*_ct_) at the temperature range from 15 K to 30 K are presented. The MgB_2_ material is characterized by the presence of two specific regions. The first region with high density, excess Mg, and rectangular MgB_2_ grains is located outside the voids surrounding them. The second region occurs inside the ceramic core, away from voids, and its chemical composition corresponds to a stoichiometric Mg to B ratio (1:2), and it is characterized by the presence of spherical grains and lower material density. A higher amount of SiC admixtures (6 at.%) causes an increase in the first region surface area. This kind of structure observation in MgB_2_ superconducting wires has never been reported previously. The transport measurements showed that higher SiC dopant leads to lower *J*_ct_ at higher temperatures and high magnetic fields. The studies showed that the point-dominant mechanism and the first region allow for obtaining high *J*_ct_ at 30 K.

## 1. Introduction

The morphology of superconducting materials significantly influences the critical parameters, particularly the transport critical current density in superconducting wires, tapes, bulks, and other applications [1,2,3,4,5,6]. The morphology of MgB_2_ superconducting materials depends on many factors, e.g., the size and purity of Mg and B grains, annealing temperature, annealing time, Mg grain shape, doping, diffusion barrier, inter-grain connections, annealing under isostatic pressure, cold isostatic pressing, and cold drawing [7,8,9,10,11,12,13,14,15,16,17]. In MgB_2_ wires produced by the powder-in-tube (PIT) method, the superconducting material morphology mainly depends on voids and cold drawing [14,15,18]. In MgB_2_ material, the morphology strongly depends on the voids created by Mg diffusion into the B layers [15,18]. The biggest problem in MgB_2_ material made by the powder-in-tube method is the random distribution of voids [18]. This makes optimizing thermal processing processes difficult and creates problems for application. The behavior of the MgB_2_ material close to the voids is essential to better understand the processes occurring in the MgB_2_ material. Studies show that the voids in the MgB_2_ material can constitute up to 25% of the volume [18]. Previous studies showed that the morphology of the MgB_2_ material in wires made by the powder-in-tube technique depends on the cold drawing process [14,15]. This process leads to the elongation of the Mg grains, the reduction of the Mg grain thickness, and the formation of B layers [15]. This makes the layered morphology of the MgB_2_ material in superconducting wires produced by the PIT method [14,15]. In addition, the voids also have an elongated shape and not a spherical shape as in MgB_2_ bulks.

Previous studies showed that the annealing process and SiC doping affect the density of the MgB_2_ material, the shape and size of the MgB_2_ grains, and the connections between the grains [19,20,21,22,23,24]. Shcherbakova et al. [19] indicate that the morphology of undoped and 10 wt.% SiC-doped MgB_2_ bulks are similar. Moreover, these studies showed that the grains’ size and shape depended on the samples’ cooling time [19]. Shorter cooling time allowed obtaining mainly small spherical grains of the order of several tens of nanometers. On the other hand, longer times led to the formation of rectangular grains of the size ∼500 nm [19]. Additionally, the results of scanning electron microscopy (SEM) and transmission electron microscopy (TEM) show that MgB_2_ grains grow faster along the c-axis rather than along the ab-plane [19]. Further results presented by Qu et al. [20] for 5 wt.% SiC-doped MgB_2_ bulks point out that an annealing temperature of 750 °C for 30 min forms grains about 0.5–0.8 μm in size, and the fine grains are less than about 200 nm in size. It has been reported that the large grains grow in two stages (starting in the solid Mg state and further growth in the liquid Mg state) [20]. On the other hand, the small grains grow in one stage in the liquid Mg state [20]. The reaction of the liquid–solid process is relatively severe and fast, causing burst nucleation and fine-grained MgB_2_ [20]. Moreover, Qu et al. [20] indicate that the SiC admixture may reduce the MgB_2_ grain size. The results presented in Ref. [20] suggest that the grain size of the undoped MgB_2_ bulks is significantly smaller than the size of the Mg grains (∼75 μm) and is only 1/3 of the diameter of the B particles (∼3 μm), indicating multiple nucleation of MgB_2_ on each B particle. The studies conducted by Zhang et al. [21] show that the excess of Mg allows for a significant increase in the density of the MgB_2_ material and indicate the spherical shape of the MgB_2_ grains. Further results suggest that excess Mg increases the number of connections between grains [21]. The results presented by Li et al. [22] for MgB_2_ wires made by the PIT technique show that the MgB_2_ material has grains close to spherical in shape and with high porosity. The additional amount of Mg leads to melting into large clusters because the additional Mg can extend the liquid reaction time [22]. The following results show that the wire with Mg_1.15_B_2_ + 10 wt.% SiC has long bar grains. Li et al. [22] indicate that this is the effect of strain that arises as a result of substituting carbon for boron [22]. The studies presented by Shi et al. [23] and Yan et al. [24] show that the MgB_2_ material has spherical grains and that annealing temperature at 1000 °C for 0.5 h does not lead to SiC decomposition [24]. Previous studies show that the SiC dopant has many advantages, such as increasing the critical current density, the irreversible magnetic field, and the upper critical field, and slightly reducing the critical temperature [25,26,27]. Studies conducted by Jung et al. [28] indicate that a nano SiC admixture can react with Mg above 600 °C and form Mg_2_Si. Li et al. [25] point out that in SiC-doped MgB_2_ material, the Mg_2_Si phase is formed first, and only then is the MgB_2_ phase formed. Studies have shown that Mg_2_Si can create strong pinning centers and increase the critical current density [29,30]. The studies showed that the high hardness of the SiC admixture located on the grain boundaries can create stresses and strains in the MgB_2_ grains during the sample cooling process [31]. The results presented by Flukiger et al. and Adamczyk et al. showed that the high density of MgB_2_ material in wires fabricated by the powder-in-tube technique allows for the increase of the transport critical current density at 4.2 K and 20 K [32,33].

The studies conducted by Li et al. [34] showed that three different regions are formed in MgB_2_ wires produced by the internal Mg diffusion (IMD) technique: MgB_2_ layer, transition region, and B-rich layer. Further studies showed that the MgB_2_ layer consists of platelet-shaped grains about 20–40 nm in width and 100–200 nm in length [34]. In contrast, the B-rich layer contains spherical grains of unreacted boron. On the other hand, the transition region is a mixture of platelet-shaped grains and spherical grains of unreacted boron [34]. These studies indicate an essential conclusion that MgB_2_ grains are plate-shaped. This is an important factor influencing the inter-grain connections. In addition, the studies of Li et al. [34] indicated a very important factor influencing the diffusion in IMD MgB_2_ wires and the density of the boron layer. These studies showed that a high density of the boron layer significantly slows down the Mg diffusion process [34]. This conclusion is also crucial for MgB_2_ wires made using the powder-in-tube technique. Chen et al. [35] show that multifilament IMD MgB_2_ superconducting wires can have both a granular distribution and be completely densified. Furthermore, the superconducting material’s morphology is shown to depend on the filaments’ size in the IMD MgB_2_ wires [35]. The results presented by Yu et al. for multifilament IMD MgB_2_ wires [36] showed that MgB_2_ layers are entirely dense, with MgB_2_ grains exhibiting a layered distribution and spherical shape. Similar results for MgB_2_ layers in IMD MgB_2_ wires were obtained by Xiong et al. [37], Guan et al. [38], and Hakan et al. [39]. On the other hand, the results presented by Ye et al. indicate that the MgB_2_ material in IMD MgB_2_ wires has a granular morphology and that doping allows for the reduction of the grain size [40]. The performed studies showed that an increase in the MgB_2_ material density in the layer leads to a rise in the transport critical current density at 4.2 K [36,37,38,39,40] and the magnetic critical current density at a temperature range from 5 K to 20 K [35].

Our research aims to indicate the influence of two-region morphology and grain shape on the transport critical current density in the range from 15 K to 30 K in PIT MgB_2_ wires. In addition, our research can also show the shape of MgB_2_ grains in the two-region morphology. Moreover, EDS analysis indicates the two-region morphology composition in PIT MgB_2_ wires. Further, the results show the influence of two-region morphology on the connection between grains, magnetoresistance, and the dominant pinning mechanism.

## 2. Materials and Methods

The one core MgB_2_ wires doped with 2 and 6 at.% SiC were made using the powder-in-tube (PIT) method in an iron shield with a fill factor of 45%. The powders with the following size and purity were used: magnesium (particle sizes: 100–200 mesh, ∼149–74 μm; 99% pure), amorphous nano boron (particle size < 250 nm; >98.5% pure Pavezyum Advanced Chemicals), and SiC (200–40 mesh grain size, 99.9% pure Sigma Aldrich). The stoichiometric 1:2 ratio mixtures of Mg and B powders with 2 and 6 at.% SiC additives were ball-milled for 3 h in an argon atmosphere. These powders were pressed in a round mold and turned into 1.00–1.50 mm thick pellets. In the next step, the same powder was filled into iron tubes for the fabrication of wires. The 250 mm long iron tubes with inner/outer diameters of 9.00 mm/12.0 mm were pre-cleaned before filling in both cases. In the last step, the cold drawing method with some intermediate heat treatment steps was applied to make the wire samples with a 1.00 mm final diameter [41]. The wires were annealed in sealed quartz ampoules filled with argon at temperatures from 630 °C to 740 °C for 40 and 720 min (Table 1).

The morphology and chemical composition of MgB_2_ wires were made using a field emission scanning electron microscope (FE-SEM) FEI Nova Nano SEM 230 (FEI Company as a subsidiary of Thermo Fisher Scientific, Hillsboro, OR, USA) integrated with an energy-dispersive X-ray spectrometer (EDAX Apollo 40 SDD, EDAX LLC, Pleasanton, CA, USA). The morphology analysis of the MgB2 material was performed using the secondary electrons method.

**Figure 1:**
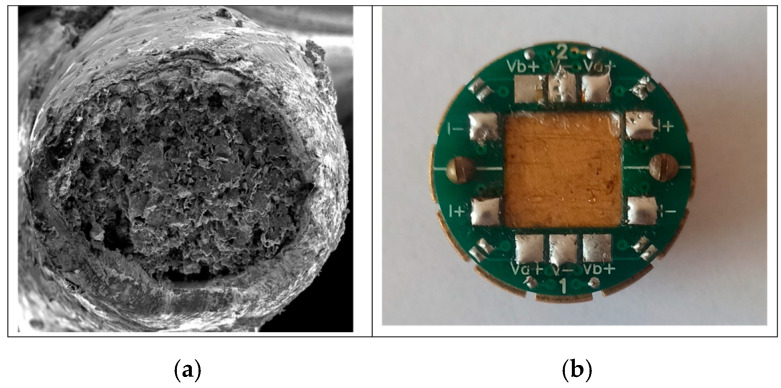
(**a**) The cross-section of MgB_2_ wire with Fe shield and (**b**) sample holder for transport measurements in PPMS.

The chemical compositional studies were carried out using an energy-dispersive X-ray spectroscopy (EDS) detector. Observations and chemical composition analyses were conducted on fractures of all MgB_2_ wires cracked at liquid nitrogen temperature (Figure 1a).

The transport critical current was measured in a perpendicular magnetic field (perpendicular to the direction of current flow through the superconducting wires) and determined based on the 1 μV/cm criteria, at temperatures from 15 K to 30 K (He vapor environment) using a cryostat equipped with a 9 T superconducting magnet (Oxford Instruments Susceptometer) and a DC current source.

Critical superconductive parameters such as irreversible magnetic field *B*_irr_, upper critical field *B*_c2_, and critical temperature *T*_c_ were measured using a physical properties measurement system, PPMS (Quantum Design, magnetic flux density up to 9 T) (Figure 1b). The *B*_irr_ was determined based on the 10% criterion, *B*_c2_ based on 90%, and *T*_c_ based on 50% of the normal resistance. The length of the MgB_2_ wires for measurements made using PPMS was 10 mm. The measurements were performed for a maximum measurement error of 2%.

## 3. Results and Discussion

### 3.1. MgB_2_ Wire Morphology

**Figure 2:**
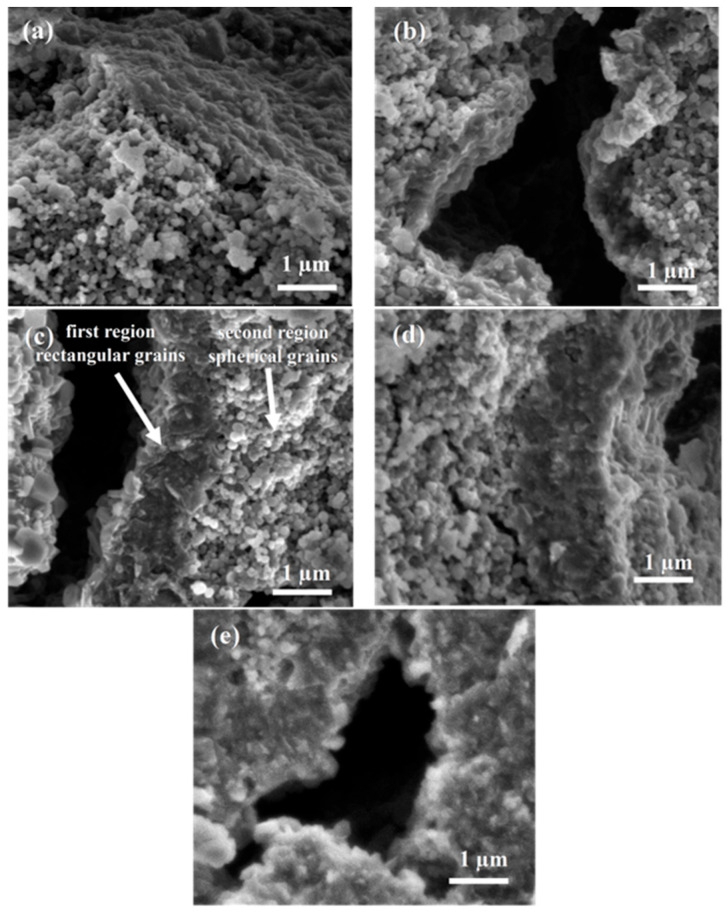
The fracture of SiC-doped MgB_2_ wires with 2 at.% SiC admixture annealed (**a**) at 650 °C for 40 min (C), (**b**) at 630 °C for 720 min (B), (**c**) at 700 °C for 40 min (D), and with 6 at.% SiC annealed (**d**) at 630 °C for 720 min (F) and (**e**) at 700 °C for 40 min (G)—low magnification.

**Figure 3:**
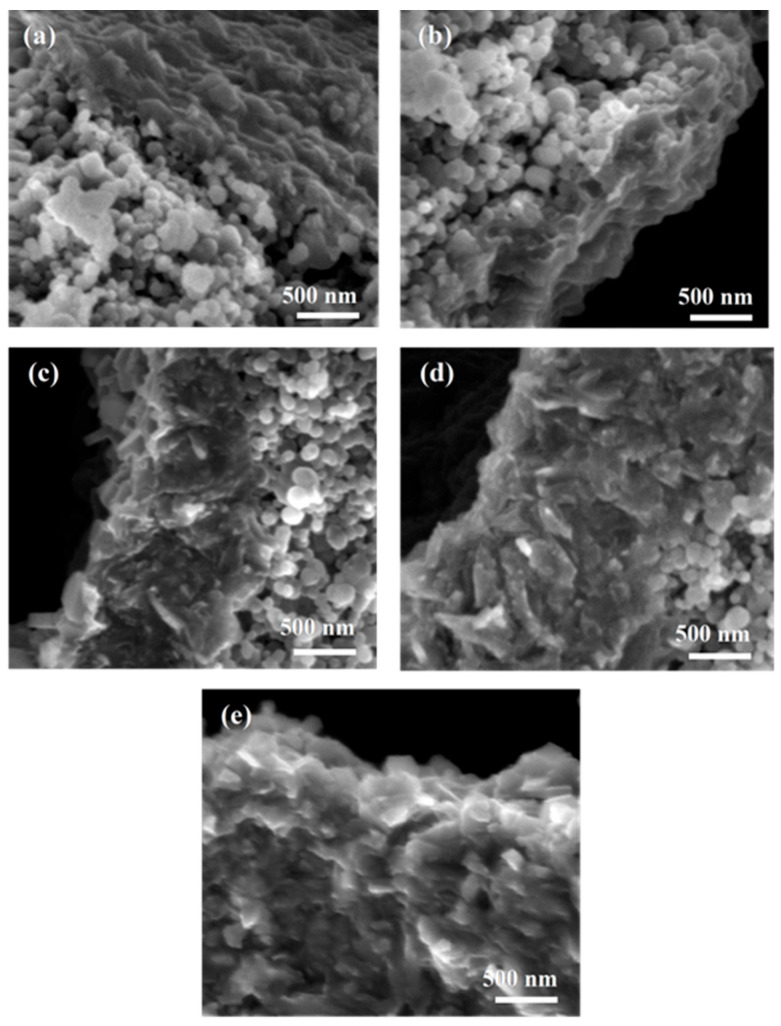
The fracture of SiC-doped MgB_2_ wires with 2 at.% SiC admixture annealed (**a**) at 650 °C for 40 min (C), (**b**) at 630 °C for 720 min (B), (**c**) at 700 °C for 40 min (D), and with 6 at.% SiC annealed (**d**) at 630 °C for 720 min (F) and (**e**) at 700 °C for 40 min (G)—medium magnification.

Morphology analysis was performed by scanning electron microscopy (SEM) using the secondary electrons (SE) method for fractured MgB_2_ wires. The SEM images show that the morphology of samples annealed at temperatures equal to or lower than the magnesium melting temperature—A, C, and E—is very similar regardless of SiC content and annealing time, and mainly consists of spherical grains (Figure 2a and Figure 3a).

Due to the similarity and to simplify the presented results, the structures for samples A and E are not included in the figures, and only sample C is presented. It is worth mentioning that sample B (Figure 2b and Figure 3b), annealed for a long time of 720 min, has a thin dense layer of 300 nm thickness outside the large voids. The only difference between samples A and C is that sample B has many large voids (over 1 µm—Figure 1b). The large voids are the effect of Mg diffusion into the boron layer [14,15,18], which indicates that the diffusion of Mg in the solid state is rather slow and requires a long time [42,43].

For samples annealed at a temperature above the melting point of magnesium, regardless of the annealing time, the formation of a two-regional morphology (Figure 2c and Figure 3c) is observed. The first region of dense material is located outside the large voids. The second region has spherical grains and a much lower density (many small voids, several tens of nanometers). This type of MgB_2_ wire morphology produced by the PIT method has not been reported yet [7,8,9,10,14,15]. The SEM results for sample D (2 at.% SiC-doped MgB_2_ wire annealed at 700 °C for 40 min) are very close to those presented for MgB_2_ wires made by the IMD method [34]. This indicates that the morphology of MgB_2_ material near large voids is similar to that of MgB_2_ superconducting material produced by the IMD method. This may indicate that the Mg diffusion mechanisms in PIT and IMD wires are similar.

The results obtained for 6 at.% SiC-doped MgB_2_ wire showed that sample E (annealed at 630 °C for 40 min, not included in the figures) has a morphology similar to sample C, and it is characterized by the presence of one region with a small number of large voids and spherical grains. This points out that the SiC dopant practically does not affect the morphology of the MgB_2_ material during the Mg solid-state reaction and short annealing time. The SEM images of sample F (630 °C for 720 min) presented in Figure 2d and Figure 3d show that increasing the annealing time causes the formation of a two-region morphology similar to sample D. In Figure 2d, we see that the thickness of the denser layer in sample F is three times greater than in sample B (Figure 2b). This indicates that the SiC dopant intensifies the formation of the dense layer in PIT MgB_2_ wires. The SEM images for sample G (Figure 2e and Figure 3e) show that this sample mainly has high-density areas and a few regions with lower-density spherical grains. Comparing the results of samples D and G, it can be indicated that the SiC dopant allows for obtaining many high-density areas. Previous studies showed that the SiC addition allows for the formation of a larger amount of the MgB_2_ superconducting phase even during the solid-state reaction of Mg [19,25]. However, they did not indicate any differences in the structure of the superconducting MgB_2_ material close to large voids.

**Figure 4:**
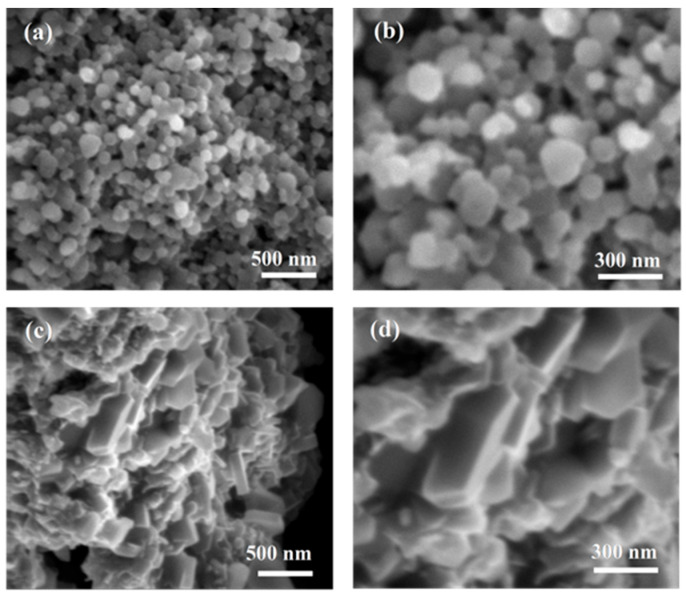
The fracture of SiC-doped MgB_2_ wires with 2 at.% SiC admixture annealed (**a**,**b**) at 650 °C for 40 min (C) as an example of spherical MgB_2_ grains, as well as (**c**,**d**) at 700 °C for 40 min (D) as an example of rectangular MgB_2_ grains—high magnification.

The structures for samples annealed at temperatures equal to and above the melting point of magnesium, observed at high magnification, are shown in Figure 4. Figure 4a,b show the 2 at.% SiC-doped MgB_2_ wire annealed at 650 °C for 40 min, characterized by the presence of spherical grains with sizes ranging from 50 to 250 nm.

On the other hand, the images in Figure 4c,d show that the dense region is formed by grains with a shape close to a rectangle with dimensions of 300 nm in length and 100 nm in width. Li et al. [34] claimed that rectangular grains are formed by the superconducting phase MgB_2,_ while spherical grains present pure unreacted boron. In addition, Li et al. show that there is a transition region between the MgB_2_ layer and the B-rich region, which is not observed in samples D, F, and G (Figure 2). Qu et al. [20] indicated that SiC-doped MgB_2_ bulks prepared by the PIT method are formed of two populations of grains: large grains of size 0.5–0.8 μm and small grains of size 200 nm. However, the distribution of large grains is random and does not form a dense region, as in samples D, F, and G. This indicates that dense regions and rectangular grains do not result from a two-stage reaction, as suggested by Qu et al. [20]. Further results presented by Shcherbakova et al. [19] for SiC-doped MgB_2_ bulk showed that rectangular grains appear in the randomly distributed MgB_2_ material and do not form a dense region. These studies indicate that rectangular grains result from long annealing times in the liquid Mg state [19]. However, our studies for samples D and G show that rectangular grains can be formed during short annealing times in the liquid Mg state in MgB_2_ superconducting wires.

### 3.2. Energy-Dispersive X-Ray Spectroscopy (EDS) Analysis

**Figure 5:**
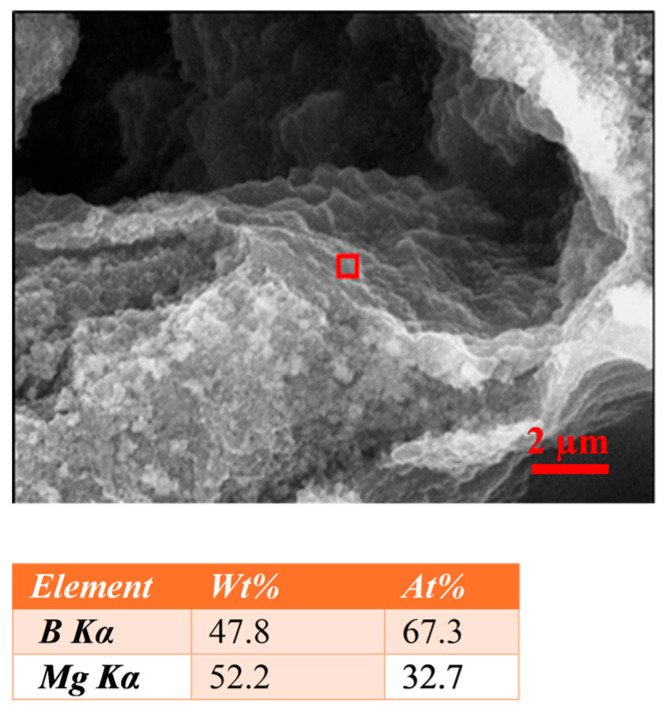
The results of the EDS analysis for sample C with 2 at.% SiC admixture annealed at a temperature of 650 °C for 40 min.

The EDS analysis performed for the samples annealed at temperatures lower than or equal to Mg’s melting point in the areas with spherical grains near large voids (Figure 5) showed that these areas contain around 32 at.% Mg and 68 at.% B, which is relatively close to the stoichiometric composition of the MgB_2_ phase [44,45]. This indicates that the areas with spherical grains consist of the MgB_2_ superconducting phase, which is the result of the reaction between nano boron and magnesium. Moreover, these results suggest that the diffusion of Mg at low temperatures (630 °C and 650 °C) in the regions with spherical grains is uniform and leads to the uniform distribution of the MgB_2_ superconducting phase. The obtained results are seemingly contradictory to the results presented by Li et al. [34]. However, it should be noted that in both works, different fabrication techniques (PIT and IMD) and different process conditions (temperature and annealing time) were used. This resulted in the formation of unreacted boron [34] or spherical MgB_2_ areas (present work).

**Figure 6:**
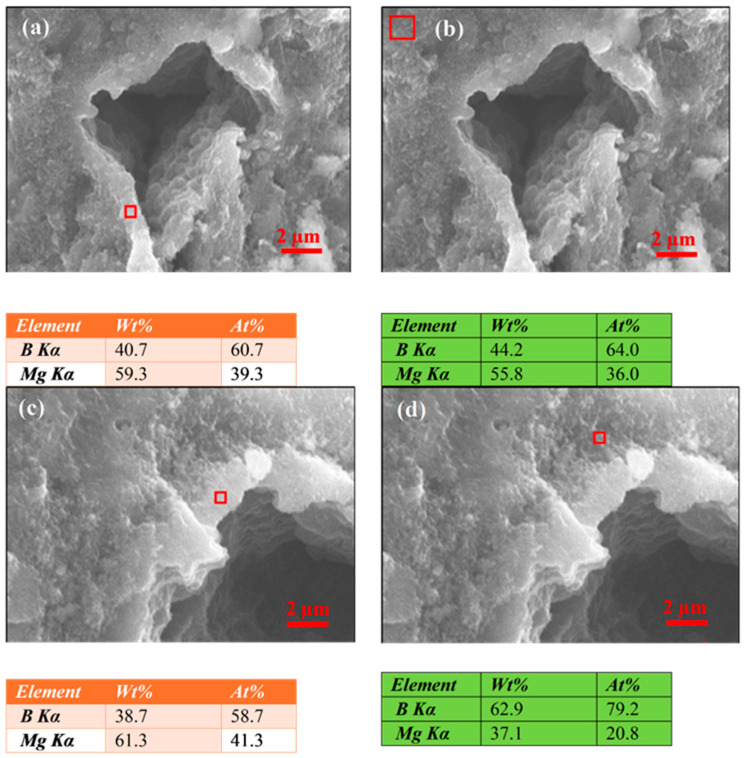
The results of the EDS analysis for (**a**) sample D—the first region with rectangular grains, (**b**) sample D—the second region with spherical grains, (**c**) sample F—the first region with rectangular grains, and (**d**) sample F—the second region with spherical grains.

The EDS analyses for samples annealed at temperatures above the Mg melting point, presented in Figure 6a,c, suggest that the first region with rectangular grains, regardless of SiC content, contains 40 at.% of Mg and 60 at.% of B. This indicates that these regions are characterized by the presence of the superconducting MgB_2_ phase separated by pure Mg. Previous transmission electron microscopy (TEM) studies showed that rectangular MgB_2_ grains occur near regions with excess Mg [41]. This may suggest that rectangular MgB_2_ grains form in regions with excess Mg. The excess Mg in the dense areas might reduce Mg in other wire regions, which may lead to a reduction in the amount of the superconducting MgB_2_ phase throughout the wire. This also explains why Mg-excess MgB_2_ wires have higher critical current density transport at 4.2 K and slightly denser MgB_2_ material morphology [44,46].

Further results in Figure 6b and d show that the regions with spherical grains have Mg content ranging from 21 at.% to 36 at.% and B content ranging from 79 at.% to 64 at.%, respectively. Such a chemical composition of the wire core, clearly different from the stoichiometric composition of MgB_2_, indicates that these regions may contain an MgB_2_ phase interspersed with pure B. The results of Susner et al. [46] show that PIT MgB_2_ wires with excess B have a lower transport critical current density at 4.2 K.

### 3.3. Transport Measurement

The measurements showed that the samples annealed at a temperature range from 630 °C to 700 °C for 40 min and 720 min with 2 at.% SiC doping have a critical temperature of 36 K (Table 1). This is close to the *T*_c_ of undoped MgB_2_ wires [41,45,47]. This indicates that carbon does not substitute boron because there is no significant reduction in *T*_c_ [47]. This also might suggest that a small amount of SiC dopant does not create stresses and strains during the cooling process [31] because this would reduce *T*_c_. The measurements for samples A and C indicate that these samples have magnetoresistance, which shows that these samples have unreacted Mg. Samples B and D do not have magnetoresistance, which suggests that these samples have a small amount of pure Mg. The transport results obtained by PPMS for samples B and D indicate that the appearance of two regions or one region does not affect the magnetoresistance. However, sample D has a large, dense layer with excess Mg.

This may indicate that excess Mg in the dense layer with rectangular MgB_2_ grains does not lead to magnetoresistance. This may suggest that the dense layer with excess Mg has MgB_2_ superconducting connections.

Further studies for 6 at.% SiC-doped MgB_2_ wires showed that an increase in the annealing temperature from 630 °C to 700 °C for an annealing time of 40 min and 720 min leads to an increase in *T*_c_ from 34.5 to 36 K (Table 1). This indicates that a large amount of SiC dopant may slow down the formation of the MgB_2_ phase. After annealing at 630 °C, the low *T*_c_ does not result from substituting C for B, because this process requires a high annealing temperature [47]. Transport measurements made by using PPMS show that the resistance of sample E in magnetic fields ranging from 0 T to 9 T increases from 1 * 10^−4^ Ω to 3.5 * 10^−4^ Ω. On the other hand, the resistance of sample F in the magnetic field range from 0 T to 9 T increases from 5 * 10^−4^ Ω to 6.5 * 10^−4^ Ω. This indicates that samples E and F have high magnetoresistance. This indicates that these wires have a large amount of unreacted Mg. However, sample G has no magnetoresistance. This indicates that sample G has a small amount of unreacted Mg. Comparing the results of samples F and G, one can see the appearance of two regions in the sample that do not affect the magnetoresistance. This indicates that magnetoresistance will only appear for large Mg particles, which are observed in samples A, C, E, and F. This also shows that the excess of Mg on the MgB_2_ superconducting grain boundaries does not create a magnetoresistance phenomenon in superconducting wires.

**Figure 7:**
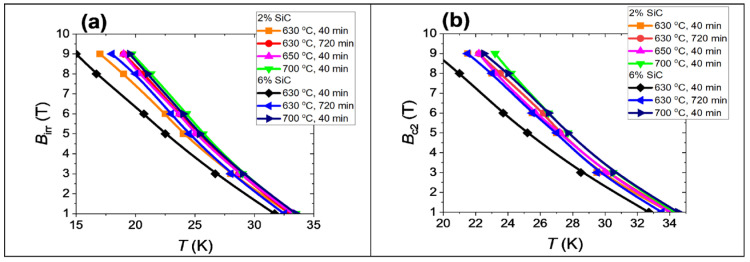
Transport measurements: (**a**) the dependence of the irreversible and (**b**) the upper magnetic field on temperature.

The measurements carried out showed that an increase in the annealing temperature from 630 °C to 700 °C for 40 min and 720 min in MgB_2_ wires with 2 at.% and 6 at.% doping leads to an increase in *B*_irr_ and *B*_c2_ (Figure 7). The measurements showed that the samples annealed in the solid state of Mg with 2 at.% SiC doping have higher *B*_irr_ and *B*_c2_ than the samples with 6 at.% doping. However, the critical parameters of the samples annealed at temperatures of liquid Mg, i.e., at or above 650 °C, with 2 at.% and 6 at.% doping are the same. The irreversible magnetic field is dependent on the pinning mechanism, influenced, among other factors, by the type of pinning centers. The upper magnetic field depends on the free path and coherence length [47]. Substituting C for B leads to a reduction of the coherence length and an improvement of *B*_c2_. Based on the above assumptions, we can conclude that the increase in *B*_irr_ and *B*_c2_ is mainly obtained due to the enhancement of the MgB_2_ superconducting phase, since we do not observe a significant increase in *B*_irr_ and *B*_c2_ between the samples with 2 at.% and 6 at.% SiC doping. In MgB_2_ wires and bulks, substituting C for B leads to increased *B*_irr_ and *B*_c2_ and a reduction in *T*_c_ [47]. Comparing the results of 2 at.% SiC-doped MgB_2_ wires with the results of 6 at.% SiC-doped MgB_2_ wires after annealing in the liquid Mg state (Figure 6), one can see that samples D and G have the same critical parameters. This indicates that carbon weakly substitutes for boron. Previous studies confirm these results [23,24]. The results of morphology (Figure 2 and Figure 3) and transport measurements (Figure 7) indicate that SiC doping accelerates the formation of a dense layer with rectangular grains but does not accelerate the formation of the optimal MgB_2_ superconducting phase with sufficient grain connections, especially in the solid state Mg, because the samples with 6 at.% SiC dopant have lower critical parameters than the samples with 2 at.% SiC.

**Figure 8:**
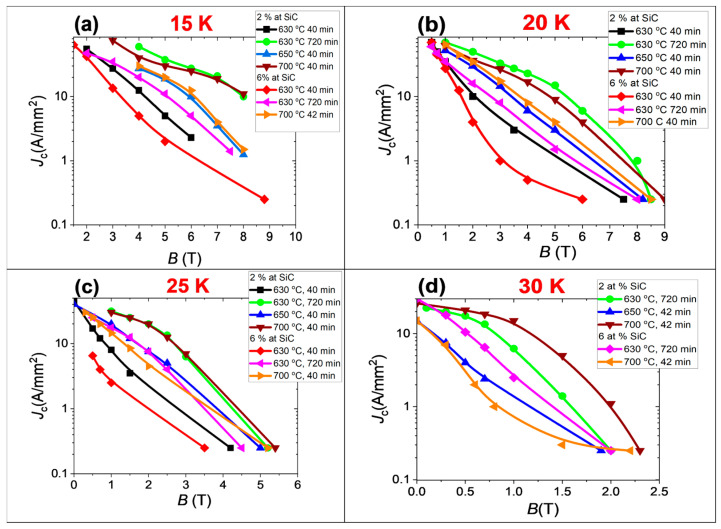
Dependence of the transport critical current density on the magnetic field at (**a**) 15 K, (**b**) 20 K, (**c**) 25 K, and (**d**) 30 K.

The measurements carried out for 2 at.% SiC-doped MgB_2_ wires indicate that an increase in the annealing temperature from 630 °C to 700 °C increases the transport critical current density (*J*_ct_) at the temperature range from 15 K to 30 K. Further results show that longer annealing time at 630 °C for 2 at.% SiC doping MgB_2_ wires significantly increases *J*_ct_ in the temperature range from 15 K to 30 K (Figure 8). Moreover, the results indicate that the *J*_ct_ of 2 at.% SiC-doped MgB_2_ wires annealed at 630 °C for 720 min (sample B) is the same as the *J*_ct_ for the 2 at.% SiC-doped MgB_2_ wires annealed at 700 °C for 40 min (sample D) in the temperature range from 15 K to 25 K. In 30 K *J*_ct_, sample D is higher than sample B (Figure 8). Further results showed that the *J*_ct_ of 6 at.% doped MgB_2_ wires also improves with an increase of the annealing temperature and annealing time at the temperature range from 15 K to 30 K. The results in Figure 8 show that a large amount of dopant (6 at.% SiC) leads to a significant reduction of *J*_ct_ in the temperature range from 15 K to 30 K.

**Figure 9:**
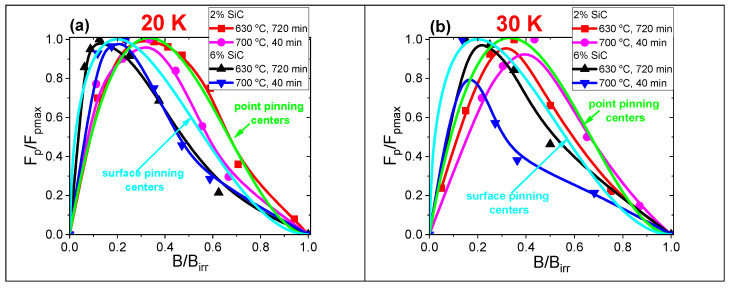
Analysis of the dominant pinning mechanism using Dew–Hughes [48] for 2 at.% and 6 at.% SiC-doped MgB_2_ wires (**a**) at 20 K and (**b**) at 30 K.

The Dew–Hughes method is used for the analysis of the dominant pinning mechanism. This method uses the following equation: f(*h*) = *h*^p^(1-*h*)^q^ [48]. In this formula, *h* is the *B*/*B*_irr_ coefficient. The parameters *p* and *q* indicate the dominant pinning mechanism. When *p* = 0.5 and *q* = 2, the sample has the surface-dominant pinning mechanism. For *p* = 1 and *q* = 2, the sample has the point-dominant pinning mechanism. The analysis of the dominant pinning mechanism performed by the Dew–Hughes method [48] indicates that the samples B and D with 2 at.% SiC dopant have a point-dominant pinning mechanism at 20 K and 30 K (Figure 9). In contrast, the samples F and G with 6 at.% SiC dopant have a surface-dominant pinning mechanism (Figure 9). The point-dominant pinning mechanism forms the regions close to the coherence length [2]. These pinning centers allow for an increase in the *J*_ct_ at middle and high magnetic fields. This leads to a higher *J*_ct_ in the wires with 2 at.% SiC dopant than in the 6 at.% SiC samples. Previous studies showed that undoped MgB_2_ wires have a surface-dominant pinning mechanism [41]. This indicates that a small amount of SiC dopant and annealing in the liquid Mg state allows the formation of normal regions with a thickness close to the coherence length/point pinning centers (matching effect [2]). The results of the Dew–Hughes analysis [48] indicate that the 6 at.% SiC dopant forms mainly surface pinning centers. Moreover, the low *J*_ct_ in the sample with 6 at.% SiC doping at high magnetic fields indicates that carbon did not substitute for boron. Such a process would cause an increase in *J*_ct_ at high magnetic fields.

Analyzing the results for 2 at.% SiC-doped MgB_2_ wires, it can be indicated that spherical grains allow the obtaining of a similar *J*_ct_ as the samples with two regions (dense and spherical grains). A positive effect of the two-region samples is seen for *J*_ct_ at 30 K. This may indicate that rectangular grains form a high-temperature MgB_2_ superconducting connection (very close to the stoichiometry of MgB_2_). Still, their number may be limited by the pure Mg at the grain boundaries. Further results suggest that spherical grains can form many MgB_2_ superconducting connections on nano-area B grains in the solid state. However, our results indicate that this process is also very efficient. Other research groups indicate that spherical grains in MgB_2_ wires lead to a high *J*_ct_ at 4.2 K [44]. The effect of rectangular MgB_2_ grains has not been studied by other research groups. The measurement results for the 6 at.% SiC doping samples showed that sample G, with a large amount of high-density area and a large amount of rectangular grains, has a higher *J*_ct_ than sample F, with a small amount of high-density area and a small amount of rectangular grains, in the temperature range from 15 K to 25 K. In contrast, at 30 K, sample F has a higher *J*_ct_ than sample G. The Dew–Hughes analysis [48] indicates that dense areas with rectangular grains create more surface pinning centers than areas with spherical grains. This is due to the large size of the rectangular grains (300 nm length). Moreover, previous studies indicate that the SiC admixture is on the grain boundaries [19,20,25,31]. This leads to the formation of surface pinning centers and a reduction in inter-grain connections, decreasing *J*_ct_.

These results indicate that rectangular MgB_2_ grains are more sensitive to grain boundary impurities than spherical grains. A small number of impurities significantly limits the number of superconducting inter-grain connections for rectangular MgB_2_ superconducting grains. Spherical impurity grains can be located in the voids and do not reduce the superconducting connection formed on the B grains.

The reduction of *J*_ct_ for large amounts of dense regions in MgB_2_ wires may also result from significant Mg deficiencies in other sample parts (Figure 6c,d). This may lead to a smaller amount of the superconducting MgB_2_ phase in superconducting wires.

Comparing the results of samples B (2 at.% SiC) and F (6 at.% SiC) after solid-state annealing of Mg, we can see that the *J*_ct_ of sample B is much higher than that of sample F. This indicates that the two-region morphology in sample F does not allow obtaining a high *J*_ct_ and that point pinning centers are necessary for a high *J*_ct_ in the temperature range of 15 to 30 K. Comparing the results of samples D (2 at.% SiC) and G (6 at.% SiC) for the Mg liquid state synthesis reaction, we see that sample G with a large number of dense areas has a much lower *J*_ct_ than sample D with fewer dense regions (Figure 8). This indicates that the high density of MgB_2_ material and rectangular grains does not lead to a high *J*_ct_ in the temperature range from 15 K to 30 K for the surface-dominant pinning mechanism. Our results may suggest that the high *J*_ct_ for MgB_2_ wires with dense regions and rectangular grains can be obtained for the point-dominant pinning mechanism without excess Mg.

Our previous studies showed that Mg^11^B_2_ wires with spherical grain morphology have significantly lower *J*_ct_ than Mg^11^B_2_ wires with dense layers [49]. However, the dense layers had Mg and B compositions close to the optimal MgB_2_ phase composition. This may suggest that dense layers with rectangular grains can achieve a high *J*_ct_ but without excess Mg, which can reduce the number of superconducting grains

## 4. Conclusions

Our studies showed that dense regions with rectangular grains and excess Mg are formed outside the large voids in SiC-doped MgB_2_ wires during the reaction in the liquid-state Mg for short annealing times and in the solid-state Mg with long annealing times. Moreover, our studies point out that a large amount of SiC doping can accelerate the formation of dense regions with rectangular grains.

Studies point out that excess Mg in the dense region reduces the amount of Mg in other parts of the wire and reduces the amount of the MgB_2_ phase in superconducting wires. Furthermore, our studies showed that rectangular grains occur only in the Mg-rich regions.

Further studies showed that samples with one region and spherical grains have a chemical composition close to the stoichiometric composition of the MgB_2_ phase.

Transport measurements performed using the PPMS measurement system showed that excess Mg in the first region (dense areas) did not lead to the appearance of magnetoresistance in the samples. Magnetoresistance appears in samples with large grains of unreacted Mg.

The results point out that 2 at.% SiC-doped MgB_2_ wires had higher *T*_c_, *B*_irr_, and *B*_c2_ than 6 at.% SiC-doped MgB_2_ wires, especially in the case of the solid-state reaction of Mg.

The studies showed that *J*_ct_ at temperatures between 15 K and 30 K is higher in 2 at.% SiC-doped MgB_2_ wires than in 6 at.% SiC-doped MgB_2_ wires. Analysis of the dominant pinning mechanism indicated that 2 at.% SiC-doped MgB_2_ wires have point-dominant pinning centers. This points out that 2 at.% SiC admixtures and thermal treatment in the liquid state of Mg allow obtaining point pinning centers in MgB_2_ wires. In contrast, 6 at.% SiC-doped MgB_2_ wires have surface pinning centers. This leads to a lower *J*_ct_ in 6 at.% SiC-doped MgB_2_ wires.

Studies indicate that a high *J*_ct_ in MgB_2_ wires with one or two regions can be achieved for a point-dominant pinning mechanism. Further measurements showed that the large amount of the first region throughout the sample with a surface-dominant pinning mechanism does not allow for high *J*_ct_ at a temperature range from 15 K to 30 K. This indicates that a high *J*_ct_ above 15 K can only be achieved for point-dominant pinning centers.

Our results indicate that a large amount of the first type of region can provide many MgB_2_ superconducting inter-grain connections. However, an excess of Mg in the first type of region reduces the number of superconducting inter-grain connections between MgB_2_ superconducting grains. This leads to a reduction in *J*_ct_.

Studies show that 2 at.% SiC-doped MgB_2_ wires with two-region morphology have the highest *J*_ct_ at 30 K. In the temperature range from 15 K to 25 K, 2 at.% SiC-doped MgB_2_ wires with one and two regions have a similar *J*_ct_. In 6 at.% SiC-doped MgB_2_ wires, the *J*_ct_ in the temperature range from 15 K to 25 K is highest for MgB_2_ wires with a large amount of the first region than for the sample with a small amount of the first region. At 30 K, the *J*_ct_ of 6 at.% SiC-doped MgB_2_ wires is highest for the samples with a small amount of the first region.

## Figures and Tables

**Table 1:** The annealing parameters and critical temperature *T*_c_ of SiC-doped MgB_2_ wires.

Sample No	Heating Temperature [°C]	Heating Time [Minutes]	The Amount of Admixture [at. %]	*T*_c_ for B = 0 T [K]
A	630	40	2	36
B	630	720	2	36
C	650	40	2	36
D	700	40	2	36
E	630	40	6	34.5
F	630	720	6	35.3
G	700	40	6	36

## Data Availability

The raw data supporting the conclusions of this article will be made available by the authors on request.

## Abbreviations

The following abbreviations are used in this manuscript:

SEM	Scanning Electron Microscopy
TEM	Transmission Electron Microscopy
EDS	Energy Dispersive Spectroscopy
PIT	Powder-in-Tube
IMD	Internal Mg Diffusion
